# Nanoparticle-Based Radiosensitization

**DOI:** 10.3390/ijms21082879

**Published:** 2020-04-20

**Authors:** Ivan Kempson

**Affiliations:** Future Industries Institute, University of South Australia, Mawson Lakes 5095, Australia; Ivan.Kempson@unisa.edu.au

Radiotherapy is a highly multidisciplinary field with respect to its foundations of research and development, and in its clinical utility. This is further evident in the field of nanoparticle radiosensitization, which introduces disciplines such as pharmaceutical sciences, colloid and surface sciences, nanomedicine and materials, the breadth of which epitomizes multidisciplinary research and has resounded in recent successes in the translational pathway of nanoparticles in this context [1,2,3]. These highly commendable successes stand as testaments to what basic sciences achieve when brought together with an applied focus in mind. Richness in the scientific diversity of this field is likewise represented by the topics covered in this Special Issue, spanning nanoparticle synthesis to delivery and underpinned by continued efforts for a greater mechanistic understanding of their function. With successful delivery of nanoparticle radiosensitizers, there is translatable scope for improving the therapeutic outcomes in treating cancer, especially where radiotherapy is limited by sparing healthy tissues in radioresistant cancers. This is exemplified, for example, in the work of Dr (MD) Kazmi et al. [4], who report on the sensitization effects of gold nanoparticles in the problematic indication of glioblastoma.

Prof Lacombe and colleagues in Yang et al. [5] described the synthesis and utility of platinum “nanoflowers”. The nanoparticles lend themselves to conjugation with targeting moieties, fluorescent labels (for tracking intracellular transport and fate) or synergistic pharmacological agents. Their data also highlight the critical role of damage localised on the nanoscale proximal to nanoparticles. This is reiterated computationally by Prof Bezak and colleagues in the work reported in Peukert et al. [6] in the computational modelling of the generation of reactive oxygen species around nanoparticles under irradiation with proton beams. Peukert et al. investigated the influence of the aggregation behaviour of nanoparticles in cells on the radiolysis yields under irradiation. While nanoparticles being in close proximity to each other can lead to increasing self-absorption of secondary electrons and lower total dose, this can still lead to localised enhancement of both dose and reactive species yield to a much greater extent than a single nanoparticle.

Sánchez et al. [7] reported on work from Prof Roux and colleagues conjugating a fluorescent label to track the intracellular fate of nanoparticles in cells. A notable result identified nanoparticles in the direct vicinity of mitochondria. Real-time in-vivo tracking of nanoparticles enables information on pharmacokinetics in individual animals. The correlation of subcellular distribution and biodistribution with outcomes in terms of both radiobiological response and toxicity will be greatly facilitated by such studies. Mechanistically, the action of nanoparticles in enhancing radiobiological effects varies greatly. From my own group, Howard et al. [8] reviewed the chemical mechanisms reported in contributing towards radiosensitization, predominantly in enhancing reactive species either by delivery of oxygen-rich material, interfacial catalysis of water dissociation or localised effects from dose enhancement. Prof Geso and colleagues, in Shahhoseini et al. [9], eluded to biological impacts caused by nanoparticles in their study of cell migration. Gold nanoparticles reduced the motility of cells and potentially altered the physical structure of cells, which also reduced their ability to adhere. The presence of nanoparticles under irradiation compounded the effect on motility, highlighting the potential of nanoparticles to fundamentally perturb the radiobiological response via cellular functions.

Dr Boateng and A.Prof. Ngwa reviewed the delivery strategies of nanoparticle radiosensitizers [10]. Critical for effective radiosensitization is preferential delivery of sensitizing agents to specifically target the desired tumour cells to assist in sparing healthy tissues. In their review, Boateng and Ngwa explored the literature in terms of delivery strategies, including targeted systemic delivery, inhalation, localized delivery via intratumoural injection, and implants loaded with nanoparticles. Szatmári et al., from Dr (MD) Lumniczky’s Lab., summarised research in using natural cellular processes for delivery of sensitizers to cells for potentiating the effects of radiation [11]. Enhancing the radiobiological response in one cell could impart enhanced bystander effects on other cells via extracellular vesicles. The signaling and opportunities in this space for enacting radiosensitization or radioprotective effects are an interesting and emerging area of research. They have further potential in acting as stealth-like carriers for sensitization agents, and tracking such vesicles would be a fascinating achievement. Nanoparticle tracking in general is of value in understanding biodistribution, pharmacokinetics and pharmacodynamics. One method is via radionuclide tracking. Radiolabeling of nanoparticles was reviewed by Prof. Jeon [12], who summarised nuclides useful for various imaging modalities and also offered therapeutic opportunities via their decay and emissions. Similar concepts were reported in work from Prof. Selomulya and Prof. Plebanski in Chakraborty et al. [13], but within the context of magnetic resonance imaging. Magnetic properties of iron oxide nanoparticles were used for imaging selective uptake by alveolar macrophages and neutrophils in the lung with the aim of assisting diagnostics and radiotherapy planning, and reducing the pulmonary complications of radiation.

Maturity in the field of nanoparticle radiosensitization research is reflected by the breadth of topics covered by four review articles comprising this overview. All reports contained within this Issue are inclusive of statements identifying the need to better understand fundamental aspects of radiosensitization. Each in their own way concur that mechanistic understanding is a driver for research in this field, and that clinical benefits are both needed and probable. This continuing translation necessitates the inclusion of numerous disciplines to encompass the fundamental aspects of radiosensitization at a basic level and in the delivery of optimised radiotherapy outcomes.
